# The Impact of Esophageal Oncological Surgery on Perioperative Immune Function; Implications for Adjuvant Immune Checkpoint Inhibition

**DOI:** 10.3389/fimmu.2022.823225

**Published:** 2022-01-27

**Authors:** Noel E. Donlon, Maria Davern, Andrew D. Sheppard, Fiona O’Connell, Margaret R. Dunne, Conall Hayes, Eimear Mylod, Sinead Ramjit, Hugo Temperley, Michael Mac Lean, Gillian Cotter, Anshul Bhardwaj, Christine Butler, Melissa J. Conroy, Jacintha O’Sullivan, Narayanasamy Ravi, Claire L. Donohoe, John V. Reynolds, Joanne Lysaght

**Affiliations:** ^1^ Cancer Immunology and Immunotherapy Group, Department of Surgery, Trinity Translational Medicine Institute, Trinity St James’s Cancer Institute, St James’s Hospital, Dublin, Ireland; ^2^ Department of Surgery, Trinity Translational Medicine Institute, Trinity St James’s Cancer Institute Trinity College Dublin, St James’s Hospital, Dublin, Ireland

**Keywords:** perioperative immunosuppression, immunotherapy, surgery, neoadjuvant, esophageal cancer, adjuvant

## Abstract

**Background:**

Immune checkpoint inhibitors (ICIs) are being investigated for their role as an adjunct in the multimodal treatment of esophageal adenocarcinoma (EAC). The most effective time to incorporate ICIs remains unknown. Our study profiles systemic anti-tumor immunity perioperatively to help inform the optimal timing of ICIs into current standards of care for EAC patients.

**Methods:**

Systemic immunity in 11 EAC patients was phenotyped immediately prior to esophagectomy (POD-0) and post-operatively (POD)-1, 3, 7 and week 6. Longitudinal serological profiling was conducted by ELISA. The frequency of circulating lymphocytes, activation status, immune checkpoint expression and damage-associated molecular patterns was assessed by flow cytometry.

**Results:**

The frequency of naïve T-cells significantly increased in circulation post-esophagectomy from POD-0 to POD-7 (p<0.01) with a significant decrease in effector memory T-cells by POD7 followed by a subsequent increase by week 6 (p<0.05). A significant increase in activated circulating CD27^+^ T-cells was observed from POD-0 to POD-7 (p<0.05). The percentage of PD-1^+^ and CTLA-4^+^ T-cells peaked on POD-1 and was significantly decreased by week 6 (p<0.01). There was a significant increase in soluble PD-1, PD-L2, TIGIT and LAG-3 from POD-3 to week 6 (p<0.01). Increased checkpoint expression correlated with those who developed metastatic disease early in their postoperative course. Th1 cytokines and co-stimulatory factors decreased significantly in the immediate post-operative setting, with a reduction in IFN-γ, IL-12p40, IL-1RA, CD28, CD40L and TNF-α. A simultaneous increase was observed in Th2 cytokines in the immediate post-operative setting, with a significant increase in IL-4, IL-10, IL-16 and MCP-1 before returning to preoperative levels at week 6.

**Conclusion:**

Our study highlights the prevailing Th2-like immunophenotype post-surgery. Therefore, shifting the balance in favour of a Th1-like phenotype would offer a potent therapeutic approach to promote cancer regression and prevent recurrence in the adjuvant setting and could potentially propagate anti-tumour immune responses perioperatively if administered in the immediate neoadjuvant setting. Consequently, this body of work paves the way for further studies and appropriate trial design is needed to further interrogate and validate the use of ICI in the multimodal treatment of locally advanced disease in the neoadjuvant and adjuvant setting.

## Introduction

The integral pillar in the multimodal treatment of locally advanced esophageal adenocarcinoma remains surgery in combination with chemotherapy alone or chemoradiotherapy, as established by the FLOT-4 trial and CROSS randomised control trials respectively (1, 2). Despite advances in treatment, the 5 year overall survival remains below 20% and is significantly impacted upon by tumor biology and nodal involvement (3–5). In addition, tumor response to neoadjuvant treatment can predict overall survival (OS), with a major pathologic response associated with a significant improvement in OS compared to no response or minor pathologic changes after neoadjuvant therapy in gastro-esophageal cancers (6). Unfortunately, only 1 in 4 of adenocarcinoma patients will achieve a complete pathological response to neoadjuvant therapies and recurrence rates remain high, with one study of 1147 patients with resected esophageal adenocarcinoma or squamous cell carcinoma demonstrating recurrences in 38% of patients, with 83% of these within the first 2 years (7). The factors responsible for this include genomic and epigenomic instability, immune evasion, angiogenesis and micro-metastatic dissemination.

Surgery, as the crucial therapeutic approach for esophageal cancer may disrupt the tumor microenvironment and may be permissive of tumor-cell shedding and production of pro-angiogenic and growth factors (8). This perioperative timeframe is postulated to be pivotal in determining long-term cancer outcomes, disproportionally with its short duration (days to weeks). It may enhance the risk of progression of pre-existing micrometastases and the initiation of new metastases - the main cause of cancer-related mortality, while simultaneously compromising immune control over residual malignant cells (9).

In the current era of surgical oncology emphasizing personalized therapy, immune checkpoint blockade (ICB) can unleash cells of the immune system that recognize and are poised to attack cancer cells. This will enhance systemic anti-tumor immunity of cells which are ordinarily held in check by molecular and cellular pathways that suppress their activation and effector functions. This potentiates anti-tumor immunity and mediates durable cancer regression for a cohort of patients, exposing a panoply of new antigens for potential immune recognition. The seminal observation that blocking the prototypical immune checkpoint receptor cytotoxic T-lymphocyte antigen-4 (CTLA-4) could mediate tumor regression in murine models (10) led to the clinical development and approval of anti-CTLA-4 as a treatment for patients with advanced melanoma (11).

The CheckMate 577 trial provides compelling evidence for adjuvant anti-PD-1 therapy (nivolumab) in patients with residual disease after multimodal therapy, with a doubling of disease-free survival compared with placebo (12). However, despite these promising results, the optimal timing for delivery of immunotherapies in the neoadjuvant and adjuvant setting to achieve a synergy between both immunostimulation and anti-metastatic effects is yet to be elucidated (13). At this time, it is also unclear whether immunotherapy has therapeutic benefit during the potentially immunosuppressive perioperative period (14). In theory however, harnessing the peri-operative period provides a therapeutic window to potentially arrest metastatic growth, enhance immune cell mediated immunity, immunological perturbations and achieve metabolic reprograming of the tumor microenvironment (TME) already initiated by neoadjuvant therapies, potentially improving long-term survival rates in patients with cancer (15, 16).

The complex biology of immune checkpoint pathways still contains many mysteries, and the full activity spectrum of checkpoint blocking drugs, and the study of the interaction of (chemo)radiation, surgery and relevant immune pathways may help fine-tune and standardize adjuvant therapy protocols for incorporating immunotherapies (8, 17). In the context of esophageal cancer, studies/trials should also extend to patients with no residual disease as they have a 30-40% risk of relapse, hence adjuvant anti-PD-1 therapy may clearly have a role in this cohort.

The purpose of this body of work is to immunophenotype the perioperative period in esophagogastric cancer patients to elucidate the potential immunostimulatory and/or immunosuppressive effects of surgery with the goal of identifying potential therapeutic windows and targets in the adjuvant setting to reduce recurrence rates and prolong survival for these patients.

## Methods

### Ethical Approval

Ethical approval was granted from the St. James’s Hospital Ethics Committee. All samples were collected with prior informed written consent for sample and data acquisition from patients attending St. James’s Hospital or from healthy donors. This study was carried out in accordance with the World Medical Association’s Declaration of Helsinki guidelines on medical research involving human subjects. Patient samples were pseudonymised to protect the privacy rights of the patients.

### Patient Cohort

Patients were recruited at the National Esophageal and Gastric Centre at St. James’s Hospital, Dublin, Ireland, which is a high-volume esophageal academic medical center. The neoadjuvant treatments for patients with locally advanced esophageal cancer are four cycles bi-weekly FLOT regimen chemotherapy and CROSS chemoradiotherapies. Prospective databases containing detailed clinical, demographic, staging, treatment, pathologic and follow-up information for all patients were maintained. This study included patients treated with curative intent only. Bloods were collected immediately pre-operatively (POD 0), days 1, 3, 7 and week 6 post-operatively.

### Whole Blood Staining for Flow Cytometric Analysis

The following fluorochrome-conjugated antibodies were added to 100µl blood at pre-optimized concentrations and incubated for 15 min at room temperature in the dark: PD-L1-PE, TIM-3-Viobright FITC, CD8-BV421 (BD Biosciences, USA), CD3-APC (Miltenyi, USA), TIGIT-PE/Cy7 and PD-1-APC/Cy7 (Biolegend, USA) and CD4-PerCpCy5.5 (eBiosciences, USA), calreticulin-AF488 (Bio-techne, USA), HMGB-1-PE, MIC-A/B-APC, (Biolegend, USA), CD45RA-PE/Cy7, CD3-PerCP, CD4-BV510, CD4-APC (Biolegend, USA), CD69-PE, CD62L-FITC, and CD27-APEeFluor780 (eBioscience, USA). Without washing red blood cells were immediately lysed using 1X red blood cell lysing solution (Biolegend, USA), according to manufacturer’s recommendations and cells were washed twice with phosphate buffered saline (PBS, Gibco, USA). Cells were resuspended in 100 µl of PBS and stained with 1 µl of zombie NIR/violet viability dye for 20 mins at room temperature in the dark which had been first diluted 1 in 10 using PBS (BioLegend, USA). Cells were washed with FACs buffer (PBS, 0.05% sodium azide (Sigma, USA) and 2% fetal bovine serum (Gibco, USA)). Cells were fixed for 15 min with 500 µl of 1% paraformaldehyde solution (Santa Cruz Biotechnology, USA) in the dark and washed with FACs buffer. Cells were resuspended in 100 µl of FACs buffer and were stored at 4°C until acquisition using the BD FACs CANTO II (BD Biosciences) using Diva software v10 and analysed using FlowJo v10 software (TreeStar Inc.).

### Collection of Serum

Whole blood was collected using vacutainer tubes suitable for collecting serum (BD Biosciences, USA). Tubes were centrifuged at 3,000 RPM for 10 minutes at room temperature and the upper serum layer was collected and stored at -80°C to be used later for experimentation.

### Quantification of Serum Immune Proteins

Serum samples were processed according to MSD (Meso Scale Discovery) multiplex protocol. To assess angiogenic, vascular injury, pro-inflammatory, cytokine, chemokine and immune checkpoint secretions a custom 54 V-plex ELISA kit and U-PLEX ELISA kit separated across 10 discrete assays was used (Meso Scale Diagnostics, USA). The multiplex kit was used to quantify the secretions of 59 analytes including CD27, CD276, CD28, CD40L, CRP, CTLA-4, Eotaxin, Eotaxin-3, FGF(basic), Flt-1, GITR, GITRL, GM-CSF, IFN-γ, IL-10, IL-12p40, IL-12p70, IL-13, IL-15, IL-16, IL-17A,IL-17A/F, IL-17B, IL-1RA, IL-1α, IL-1β, IL-2, IL-21, IL-22, IL-23, IL-27, IL-31, IL-4, IL-6, IL-8, IL-9, IP-10, LAG3, MCP-1,MCP-4, MDC, MIP-1α, MIP-1β, MIP-3α, OX40, PD1, PD-L1, PD-L2, PIGF, TARC, Tie-2, TIGIT, TIM-3, TNF-α, TNF-β, TSLP, VEGF-A, VEGF-C, VEGF-D. All assays were run as per manufacturer’s recommendation, an overnight supernatant incubation protocol was used for all assays except Angiogenesis Panel 1 and Vascular Injury Panel 2 which were run according to the same day protocol. Analyte concentrations were calculated using Discovery Workbench software (version 4.0). Values outside the kits limit of detection were not reported.

### Statistical Analysis

Data were analysed using GraphPad Prism 9 (GraphPad Prism, San Diego, CA, USA). To compare differences between paired treatments of patient samples, a Wilcoxon signed rank test was conducted. For the MSD data One-Way Repeated Measure Analysis of Variance with *post-hoc* Tukey was performed. Statistical significance was determined as p ≤ 0.05. Corrplots were devised by Spearman correlations and attributed as positive and negative correlations and significance based on the following values - 0.4-0.59 moderate, 0.6-0.79 strong and 0.8-1 very strong.

## Results

### Patient Demographics

A total of 11 adenocarcinoma patients for all time points were included for analysis. 73% (n=8) were male and the median age was 66.72 (SD 10.26). There was one R1 resection, 73% of tumors were poorly differentiated and 91% of patients had at least one adverse feature of tumor biology (3). 10 patients underwent neoadjuvant therapy with 7 patients receiving FLOT chemotherapy, of which 6 patients tolerated all pre-operative cycles. One patient had FOLFOX and 2 patients has CROSS chemoradiotherapy (**Table 1**). Four patients in the cohort have had recurrence thus far with two local and two systemic. 80% of patients had a tumor regression grade (TRG) of 3 or greater as defined by Mandard. In terms of final histology, 63% were T3, and 45% were node positive. For complications, 5 patients had Grade III or greater as defined by Clavien Dindo. There were no post-operative deaths.

**Table 1:** Treatment modalities, cycles tolerated, tumor recurrence.

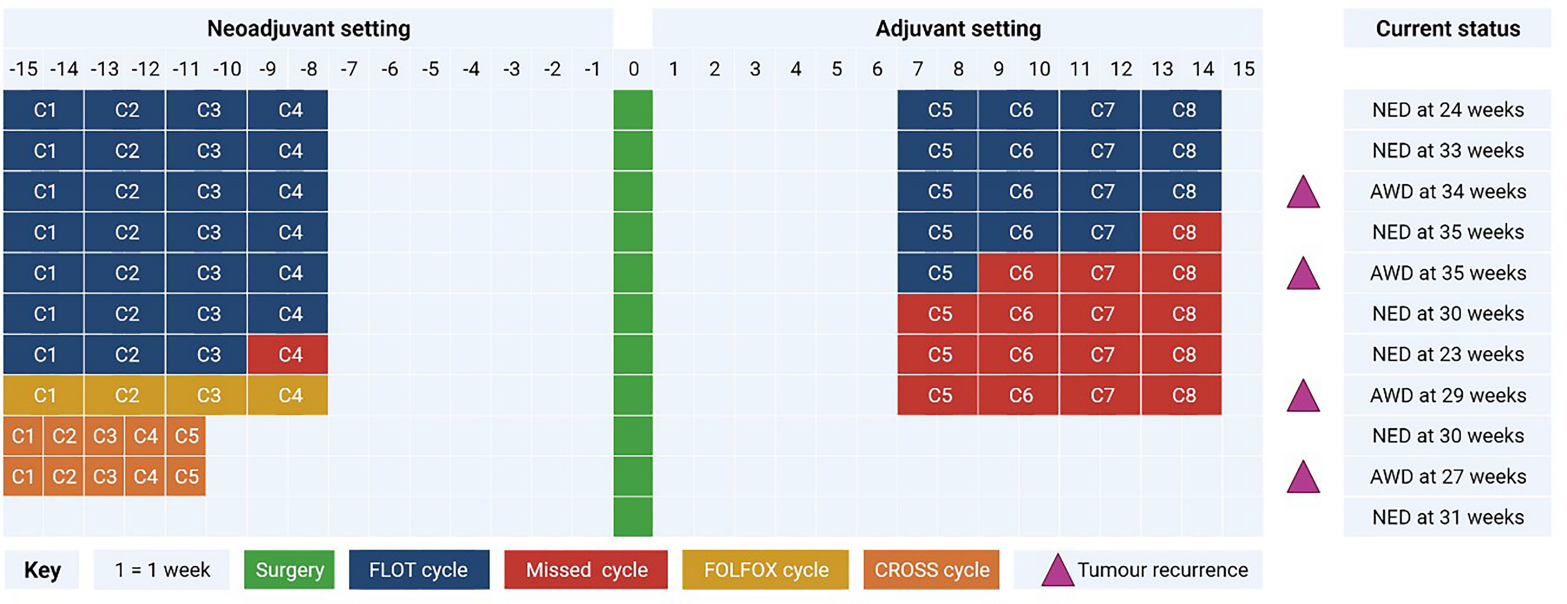	

NED, No evidence of disease; AWD, Alive with disease.

The majority of the patients who experienced complications while Grade III or greater required technical intervention. Four patients required interventional radiology, two for pleural effusions two for drainage of a chylothorax and one return to theatre for a chyle leak. Two of these patients had severe sepsis and these two patients had recurrence. They also had the highest levels of VEGF-A and VEGF-C.

### Alterations in the Circulating T Cells in the Post-Op Setting

**Figure 1:**
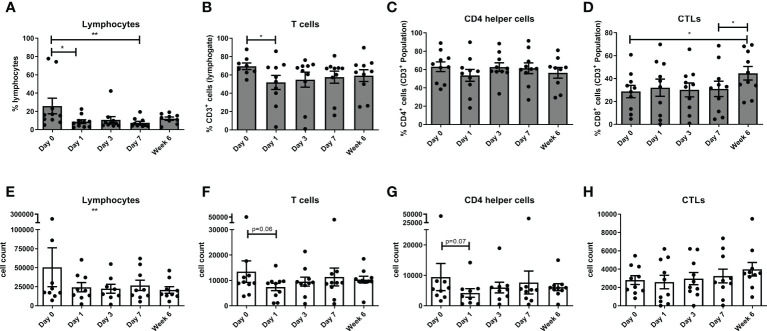
Circulating levels of lymphocytes decrease with a concomitant increase in circulating CTLs post-operatively. The percentage of circulating lymphocytes **(A, E)**, T cells **(B, F)**, CD4 T helper cells **(C, G)** and CTLs **(D, H)** were determined by flow cytometry in OAC patients on the day of tumor resection prior to surgery (Day 0) and on post-operative days 1, day 3, day 7 and week 6 (n=11). **(A–D)** displays the frequency of cell types and **(E–H)** depicts the cell counts for each cell type in peripheral circulation at each longitudinal time point. Paired, non-parametric t-test *p<0.05 and **p<0.01.

There was a decrease in total lymphocytes in the immediate post-operative phase at day 1 (p<0.05) and week 1 (p<0.01), with a decrease in the CD3 compartment at day 1 (p<0.05) when compared to basal levels. There was an increase in CD8 cytotoxic lymphocytes at week 6 (p<0.05) when compared to basal levels (**Figure 1**).

**Figure 2:**
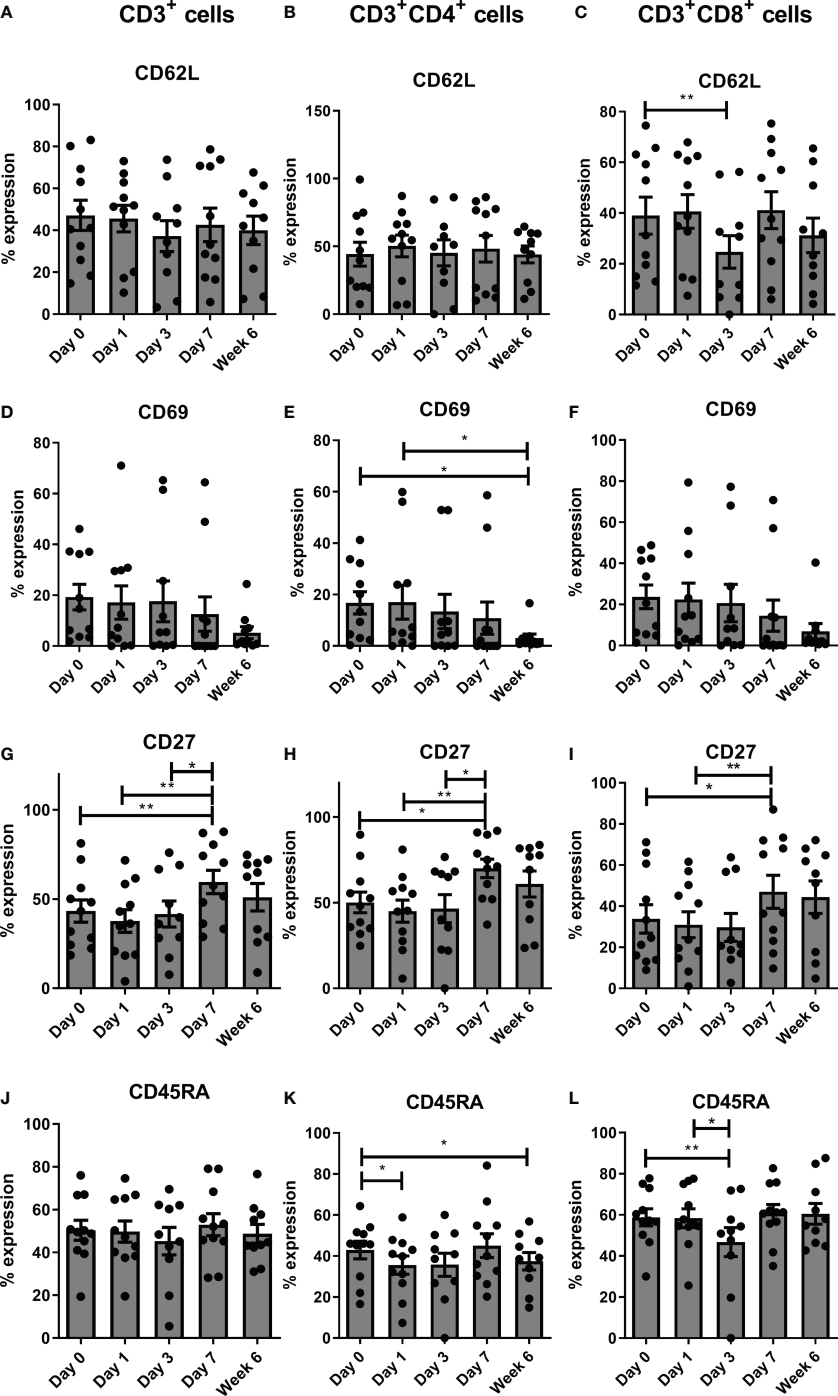
Frequencies of circulating CD27^+^ T cells increase sequentially in the immediate post-operative period. Expression of the following T cell activation markers CD62L **(A–C)**, CD69 **(D–F)**, CD27 **(G–I)** and CD45RA **(J–L)** were assessed on circulating CD3^+^, CD3^+^CD4^+^ and CD3^+^CD8^+^ cells by flow cytometry in OAC patients on POD 0, 1, 3, 7 and 6 weeks post-surgery 6 (n=11). Paired, non-parametric t-test *p<0.05 and **p<0.01.

There was a significant decrease in expression of CD62L on CD3^+^CD8^+^ cells from pre-operative levels to POD 3 (p<0.01), a significant decrease in CD69 on CD3^+^CD4^+^ cells from POD 0 to post operative week 6 (p<0.05) and from POD 1 to post op week 6 (p<0.05), and a significant increase in CD27 on CD3^+,^ CD3^+^CD4^+^, and CD3^+^CD8^+^ cells from pre-operative levels to POD 7 (P<0.01), POD 1 to POD 7 (P<0.01) and from POD 3 to POD 7 (P<0.05) (**Figure 2**). There was a significant decrease in CD45RA on CD3^+^CD4^+^ cells from POD 0 to POD 1 (p<0.05) and week 6 post-operatively (p<0.05) and a significant decrease in CD45RA on CD3^+^CD8^+^ cells on day 3 post-operatively (p<0.01) compared to pre-operative levels and also from POD 1 to POD 3 (P<0.05) (**Figure 2**).

**Figure 3:**
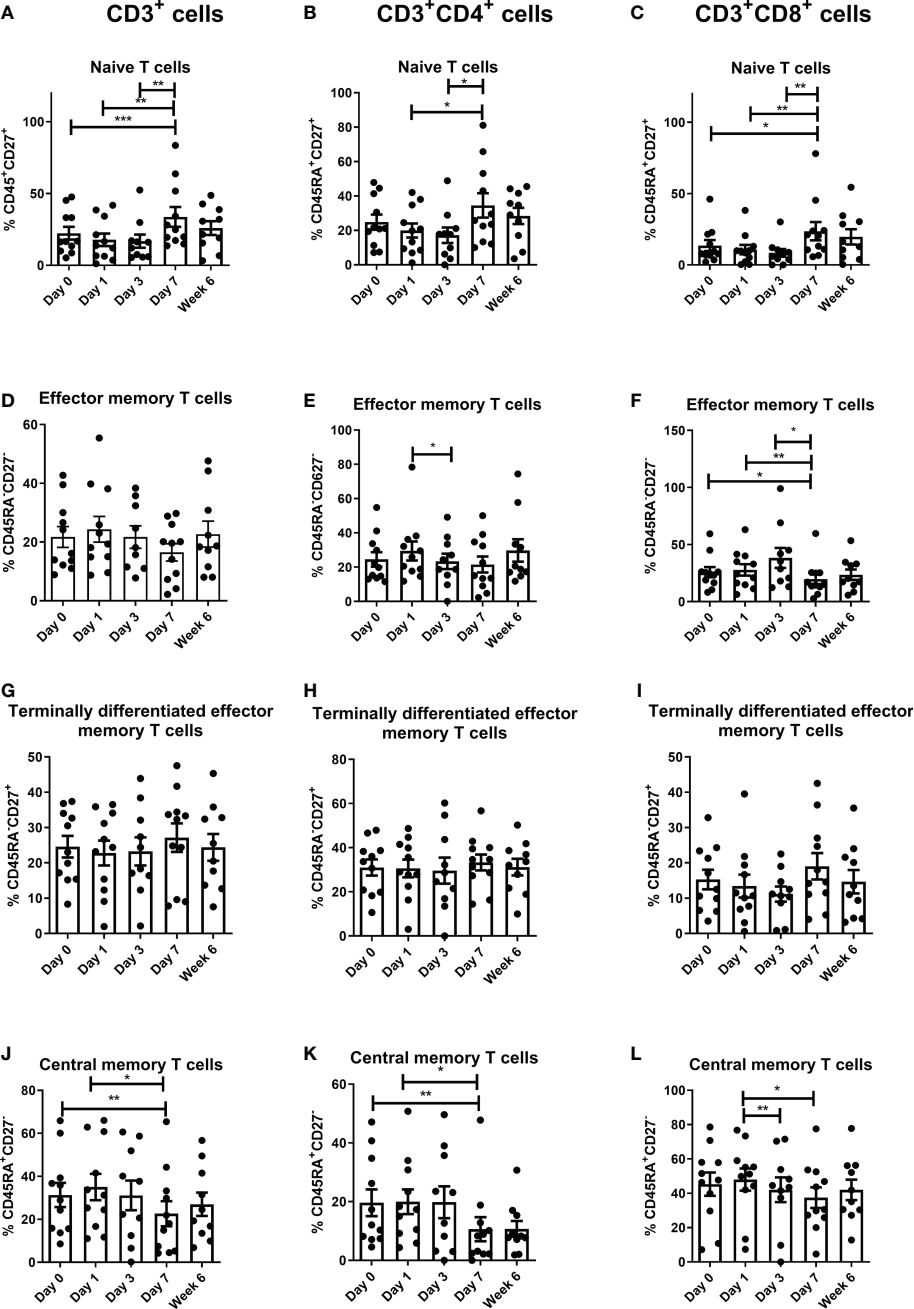
Frequencies of central memory T cells decrease and naïve T cells increase in circulation in the immediate post-operative period. The percentage of peripheral blood naïve [CD45RA^+^CD27^+^ **(A–C)**], effector memory [CD45RA^-^CD27^-^ **(D–F)**] terminally differentiated effector memory [CD45RA^-^CD27^+^ **(G–I)**] and central memory [CD45RA^+^CD27^-^ **(J–L)**] CD3^+^ cells, CD3^+^CD4^+^ and CD3^+^CD8^+^ cells in OAC patients was determined on POD 0, 1, 3, 7 and 6 weeks post-surgery by flow cytometry (n=11). Paired, non-parametric t-test *p<0.05, **p<0.01 and ***p<0.001.

There was a significant increase in naïve T cells in the peripheral circulation in the CD3 compartment from pre-operative levels to POD 7 (P<0.001), POD 1 to POD 7 (P<0.01) and POD 3 to POD 7 (P<0.01) (**Figure 3**). There was also a significant increase in CD3^+^CD4^+^ naïve T cells from POD 1 to POD 7 (P<0.05) and from POD 3 to POD 7 (P<0.05) (**Figure 3**). Finally, there was a significant increase in CD3^+^CD8^+^ naïve T cells at POD 7 compared to preoperative levels (p<0.05), from POD 1 to POD 7 (P<0.01), and from POD 3 to POD 7 (P<0.01) (**Figure 3**). In contrast, there was a significant decrease in CD3^+^CD4^+^ effector memory T cells from POD 1 to POD 3 (P<0.05) and also a significant decrease in CD3^+^CD8^+^ effector memory T cells from pre-operative levels to POD 7 (P<0.05), from POD 1 to POD 7 (P<0.01), and again from POD 1 to POD 7 (P<0.05) (**Figure 3**). There was a significant decrease in CD3^+^ and CD3^+^CD4^+^ central memory T cells from pre-operative levels to POD 7 (P<0.01) and from POD 1 to POD 7 (P<0.05), and CD3^+^CD8^+^ central memory T cells from POD 1 to POD 3 (P<0.01) and from POD 1 to POD 7 (P<0.05) (**Figure 3**).

**Figure 4:**
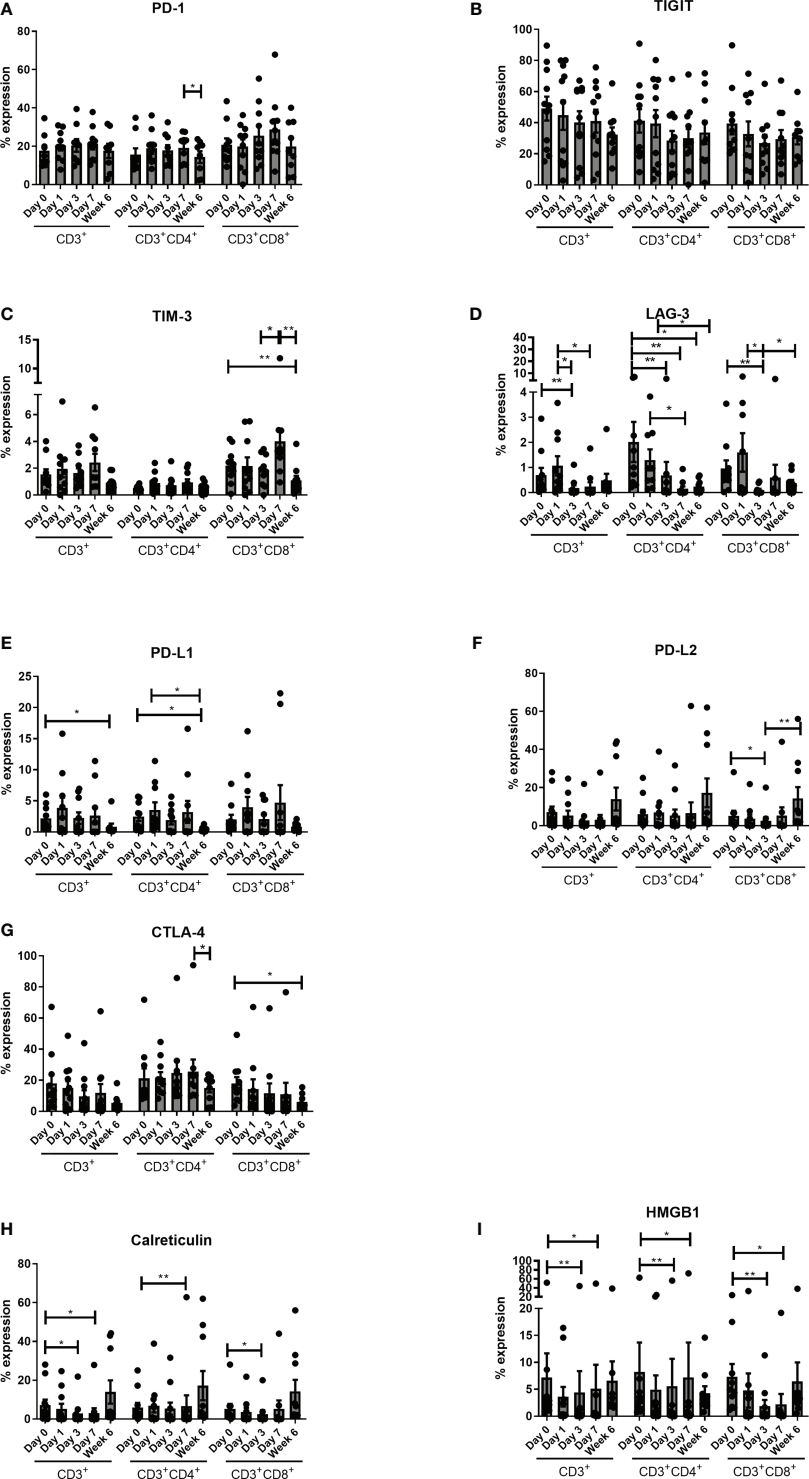
Expression of circulating CD4^+^CTLA-4^+^ cells decrease 6 weeks post-operatively. Expression of inhibitory immune checkpoints PD-1 **(A)**, TIGIT **(B)**, TIM-3 **(C)**, LAG-3 **(D)** CTLA-4 **(E)**, PD-L1 **(F)** and PD-L2 **(G)** and DAMPs HMGB1 **(H)** and Calreticulin **(I)** were assessed on the surface of circulating CD3^+^, CD3^+^CD4^+^ and CD3^+^CD8^+^ cells in OAC patients on POD 0, 1, 3, 7 and 6 weeks post-surgery by flow cytometry (n=11). Paired, non-parametric t-test *p<0.05 and **p<0.01.

There was an overall decrease in immune checkpoint expression globally, with a significantly reduced expression of CD3^+^CD4^+^PD-1^+^ from POD-7 to week 6 (p<0.05). There was a significant decrease in expression of TIM-3 in the CD3^+^CD8^+^ compartment from preoperative to post-op week six (p<0.01), from POD 3 to POD 7 (P<0.05) and from POD 7 to post op week 6 (p<0.01). There was a significant decrease in LAG-3 expression across all three T cell compartments post operatively with a decrease from POD 0 to POD 3 (P<0.01), POD 1 to POD 3 (P<0.05), and from POD 1 to POD 7 (P<0.05) in the CD3^+^ compartment. There was a significant decrease in CD3^+^CD4^+^LAG-3^+^ from preoperative levels to POD 3 (P<0.01), POD 7 (P<0.01) and week 6 (p<0.05), with a decrease from POD 1 to POD 7 (P<0.05) and week six (p<0.05). Similarly, there was a significant decrease in CD3^+^CD8^+^LAG-3^+^ from POD 0 to POD 3 (P<0.01) and from POD 1 to POD 3 (P<0.05). For PD-L1, there was a significant reduction in expression on CD3^+^ cells from pre-operative levels to week 6 (p<0.05) and CD3^+^CD4^+^ cells from pre-operative levels to post-operative week 6 (p<0.05) and from POD 1 to post-operative week 6 (p<0.05), whereas there was a significant reduction in PD-L2 expression in the cytotoxic T cell compartment from pre-operative levels to POD 3 (P<0.05) and from POD 3 to post-operative week 6 (p<0.01). There was significantly less CTLA-4 expression by CD3^+^CD4^+^ from POD 7 to post op week 6 (p<0.05) and CD3^+^CD8^+^ cells from preoperative levels to post op week 6 (p<0.05) (**Figure 4**).

There was a significant decrease in the expression of DAMPS (Calreticulin and HMGB1) across all three compartments from preoperative basal levels. There was a decrease in CD3^+^ Calreticulin from preoperative levels to POD 3 (P<0.05), and POD 7 (P<0.05), with a decrease in CD3^+^CD4^+^ Calreticulin from pre-operative levels to POD 7 (P<0.01) and again similarly in CD3^+^CD4^+^CD8^+^ from preoperative levels to POD 3 (P<0.05). In terms of HMGB1 there was a significant decrease in CD3^+^, CD3^+^CD4^+^ and CD3^+^CD8^+^ positivity from pre-operative levels to POD 3 (P<0.01) and POD 7 (P<0.05). Similarly, there was a significant decrease in Calreticulin from pre-operative levels to POD 7 (**Figure 4**).

### Multiplex Dataset

There were a total of 59 analytes n the ELISA dataset across the study timepoints with variability in trends across the data in terms of expression and this is represented in **Table 2**.

**Table 2:** Multiplex ELISA data, with trends in mean overall expression (green is increasing, red is decreasing and white is no change compared with POD 0) for cytokines, chemokines, soluble checkpoints, inflammatory and angiogenic markers for the entire cohort.

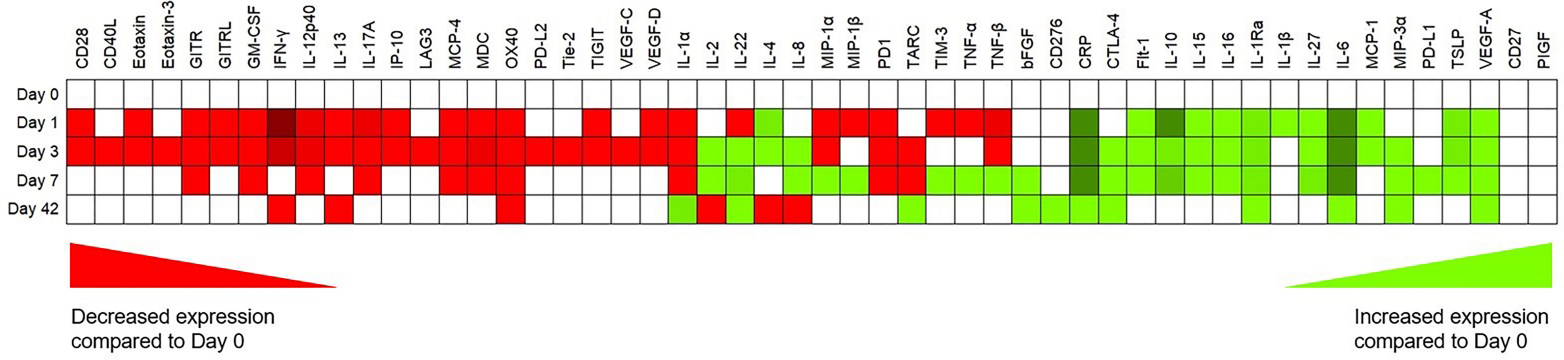	

**Figure 5:**
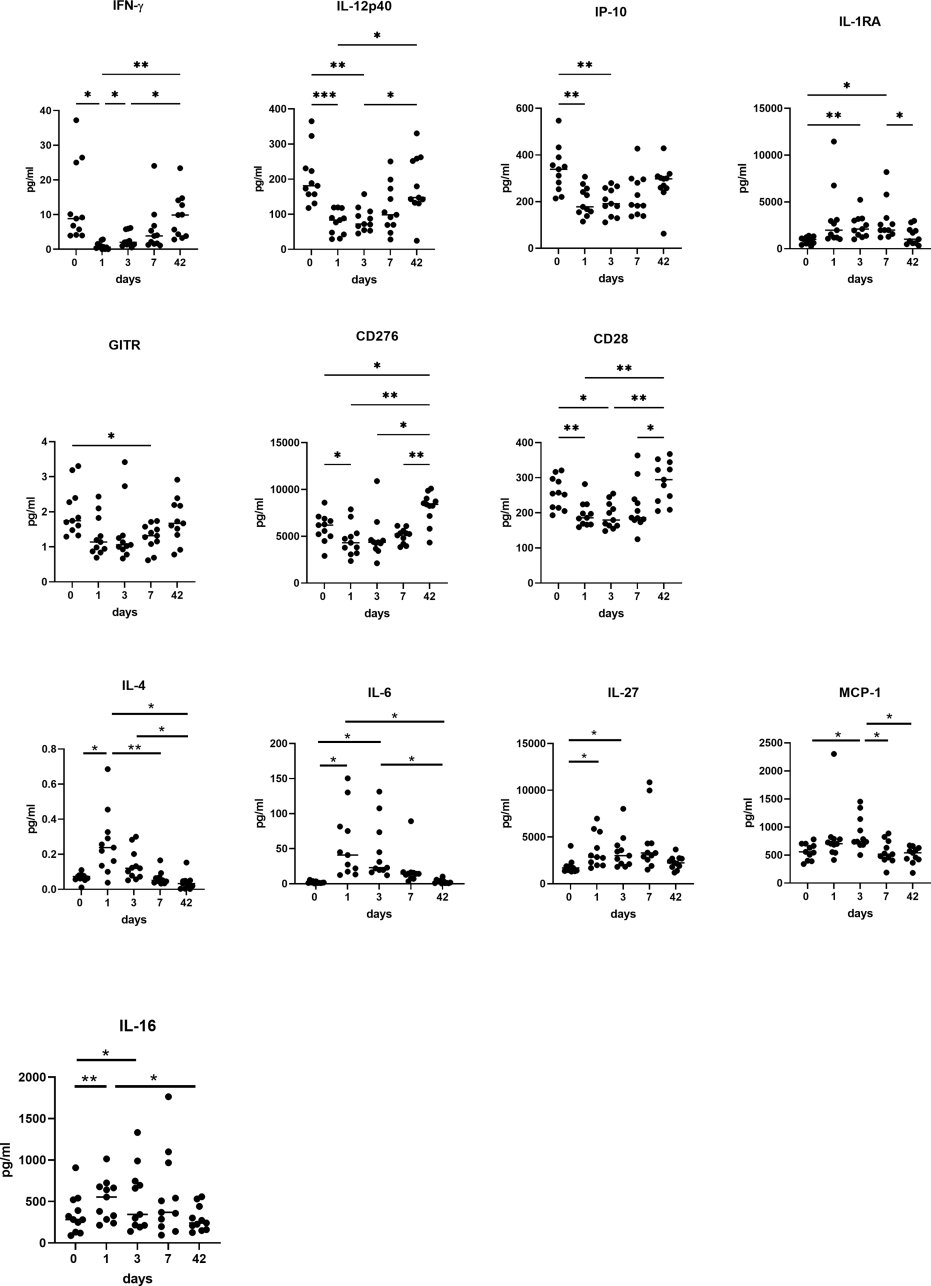
A switch from Th1 to Th2 immunity is observed in systemic circulation in OAC patients in the immediate post-operative setting. Preoperatively, POD 1, 3 and 7 and week 6 serums from OAC patients (n=11) were screened for a panel of soluble Th1 mediators (IFN-γ, IL-12 p40, IL-1RA and IP-10) and soluble co-stimulatory molecules (GITR, CD276, CD28), and Th2 mediators (IL-4, IL-6, IL-27) and soluble chemokine ligands and pro-inflammatory mediators responsible for promoting pro-tumor immune cells (MCP-1 and IL-16) by ELISA. *p<0.05, **p<0.01, ***p<0.001.

There was a significant decrease in the Th1 mediators IFN-γ, IL-12p40, and an increase in IL-1RA, which acts as an immunosuppressive mediator (**Figure 5**). There was a significant decrease in IFN-γ and IL-12p40, IP-10 and IL-1RA at POD 1 (p<0.05), POD 3 (P<0.01) and POD 7 (P<0.05), with an increase in IFN-γ at post op week 6 compared to POD 1 (P<0.001) (**Figure 5**). The co-stimulatory immune checkpoints GITR, CD276, CD28 and CD40L decrease in the immediate post-operative period before returning to pre-operative levels. The levels of GITR, CD276 and CD28 decrease at POD 1 (P<0.05) and POD 3 (P<0.01) compared to pre-operative levels with a significant increase in expression of these markers at week 6 compared to POD 3 (P<0.001).

On the contrary there was an increase in the Th2 mediators IL-4, IL-6, IL-27, soluble chemokine ligands and pro-inflammatory mediators responsible for promoting pro-tumor immune cells; MCP-1, and IL-16 (**Figure 5**). There was a significantly higher expression of IL-4 on POD 1 compared to post op week 6 (p<0.01), and POD 3 compared to POD 7 (P<0.05) and week 6 (p<0.001). There was a significantly higher expression of IL-6 on POD 1 compared to pre-operative levels (p<0.001), POD 7 (P<0.05) and week 6 (p<0.001). There was a significantly higher expression of IL-6 at POD 3 (p<0.001) compared to week 6 and pre-operative levels (p<0.001) and POD 7 compared to basal levels (p<0.01) and week 6 (p<0.05). There was also a significantly higher expression of IL-27 at POD 1 (P<0.01), POD 3 (P<0.001) and POD 7 (P<0.001) compared to basal levels. There was a significantly higher expression of MCP-1 at POD 3 compared to POD 7 (P<0.01), week 6 (p<0.05) and POD 0 (p<0.05). with a higher expression of IL-16 at POD 1 (P<0.01) and POD 3 (P<0.01) compared to basal levels with a significant reduction at week 6 compared to POD 1 (P<0.01) and POD 3 (P<0.01).

**Figure 6:**
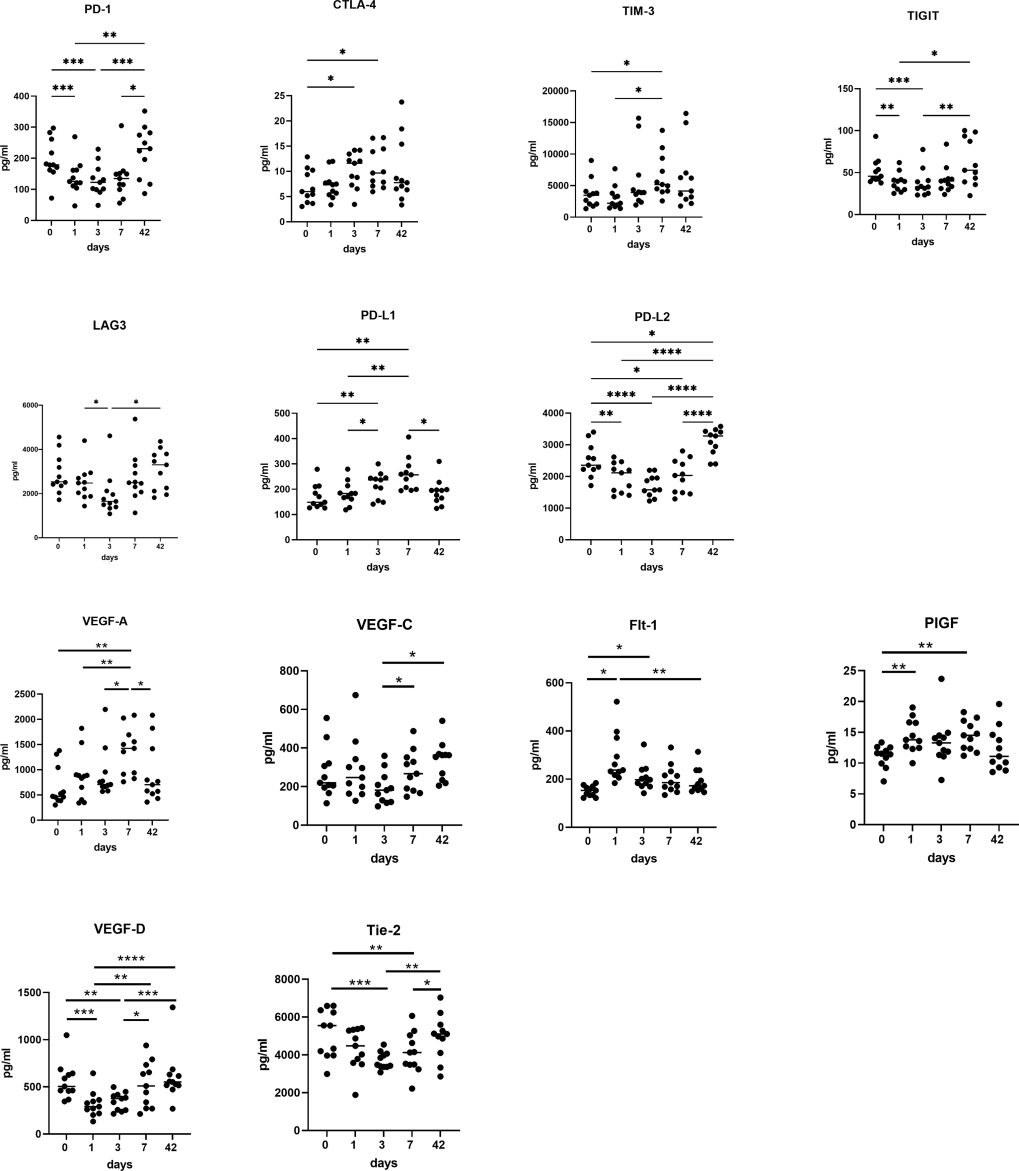
Circulating levels of pro-angiogenic and pro-metastatic factors increase in the immediate post-operative setting. POD 0, 1, 3 and 7 and week 6 serums from OAC patients (n=11) were screened for a panel of soluble inhibitory immune checkpoints PD-1, CTLA-4, TIM-3, TIGIT, LAG-3, PD-L1 and PD-L2 and a panel of soluble pro-angiogenic and pro-metastatic mediators VEGF-A, VEGF-C, PIGF, FLT-1, VEGF-D and Tie-2 by ELISA. *p<0.05, **p<0.01, ***p<0.001, ****p<0.0001.

There were contrasting trends in the soluble checkpoints with an increase in PD-L1, CTLA-4 and TIM-3 immediately post operatively, whereas PD-1, PD-L2, TIGIT and LAG-3 all reduced significantly in the immediate post-operative setting (**Figure 6**). There was a significant increase in PD-L1 (p<0.01), CTLA-4 (p<0.05), and TIM-3 (p<0.05) at POD 7 compared to pre-operative levels. There was an increase in PD-L1 at POD 3 compared to pre-operative levels (p<0.01). There was a significant increase in TIM-3 levels at POD 7 (P<0.01) and week 6 (P<0.01) compared to POD 1. There was a significant decrease in expression of PD-1 (p<0.01), PD-L2 (p<0.01), and TIGIT (p<0.05) at POD 1, POD 3 and POD 7 compared to pre-operative levels with a significant increase in expression of these markers from POD 1 to post op week six (p<0.01). There was a significant increase in PD-1, PD-L2, TIGIT and LAG-3 from POD 3 to week 6 (p<0.01).

There was a significant increase in circulating levels of pro-angiogenic and pro-metastatic markers VEGF-A, VEGF-C, PIGF and Flt-1, with a decrease in VEGF-D and Tie-2, with a return to pre-operative levels at week 6 (**Figure 6**). There was a significantly higher expression of VEGF-A and VEGF-C at POD 3 (P<0.05) and POD 7 (P<0.001) compared to pre-operative levels and a significantly lower expression of VEGF-A at week 6 compared to POD 7 (P<0.01). PIGF was significantly higher at POD 7 compared to preoperative levels. (p<0.05), with FLT-1 significantly higher at POD 1 (P<0.001) and POD 3 (P<0.01) compared to basal levels. VEGF-D was significantly lower at POD 1 (P<0.001) and POD 3 (P<0.01) compared to basal levels. It was however, significantly higher at week six compared to POD 3 (P<0.01) and POD 1 (P<0.001). Tie 2 was significantly lower at POD 3 (P<0.001) and POD 7 (P<0.01) compared to preoperative levels.

## Discussion

This seminal study is the first of its kind to longitudinally profile systemic anti-tumor immunity and circulating pro-metastatic mediators in esophageal adenocarcinoma patients perioperatively. A number of issues remain with the current multimodal treatment for these patients, namely, poor treatment responses to the current gold standards, upstaging after neoadjuvant treatment, tolerance and toxicities. In addition to this, poor uptake in the adjuvant setting secondary to the significant morbidity of the surgical deconditioning of the patient and also an understandable reluctance to use therapies post operatively which have had such poor regression results neoadjuvantly (18).

The decrease in circulating lymphocytes in the immediate post-operative phase followed by the increase in cytotoxic lymphocytes at week 6 is intriguing in the context of the checkmate-577 trial data, as they found that the timing of adjuvant nivolumab after surgery has an impact on treatment efficacy, specifically >10 wk compared with < 10 wk, with Hazards ratios (HR) (95% CI) of [0.66 (0.52-0.84)] and [0.84(0.57-1.22)], respectively. Although a better physical recovery as the authors suggest may be key, a direct immunologic explanation such as increased circulating CD8^+^ cells 6 weeks post-surgery from our study may be plausible, allowing more time for recovery of the patient’s immune system post-chemo(radio)therapy treatment. However, the increased frequency of circulating CTLs 6 weeks post-surgery, might also reflect a build-up of CTLs in circulation in parallel with reduced homing of CTLs to the site of tumor excision, due to the removal of tumour-derived chemotactic signalling. This highlights a possible double-edged sword of surgery that not only removes immunosuppressive mediators but also anti-tumor factors which might contribute to immune escape of residual pro-metastatic tumor cells and chemo(radio) resistant tumor cells that remain post-treatment and resection.

Within the present study, a significant decrease in CD3^+^CD4^+^CD69^+^ cells was observed at week six, potentially due to the removal of the immunosuppressive tumour. In addition to this, T_RM_ cells express inhibitory checkpoint molecules and may serve as potential targets for cancer immunotherapy (19). It has also been demonstrated that an increase the early memory T cells including naïve T cells and effector cells is essential for efficient tumor killing especially for adoptive cancer immunotherapy (20, 21). Furthermore, T cells with a naïve phenotype from TCR transgenic mice demonstrated enhanced anti-tumor activity following adoptive cell transfer compared with their mature T cell counterparts (22). In the current study, there was an increase in naïve T cells and a decrease in effector memory and central memory T cells in the immediate perioperative period and may reflect the adaptation of the host immune system to overcome the immunosuppressive milieu or indeed that the immunosuppressive environment has been removed.

The expression of the inhibitory immune checkpoint PD-1 on circulating T cells was highest in the immediate post-operative setting and significantly decreased by week 6 and this is important in the context that programmed death-1 (PD-1) upon interaction with its ligand PD-L1, plays cardinal roles for induction of immune evasion in cancer cells (23). Similar trends were found for surface expression of CTLA-4, TIM-3, LAG-3, PD-L1 and PD-L2 on T cells with no significant change in TIGIT expression on the surface of T cells and suggests ICB as a target in the perioperative period.

There was a significant effect of surgery on the levels of circulating soluble immune checkpoints with a significant decrease in circulating levels of soluble PD-1 and LAG-3, in immediate post op setting, which return to baseline 6 weeks post operatively, with similar findings for PD-L2, TIGIT, and TIM-3 and this may reflect removal of their source of production through tumor excision and tumor-infiltrating immune cells. Soluble immune checkpoints may be a good or poor prognostic indicator depending on the soluble immune checkpoint in question, however, little is known about their function in particular for soluble TIGIT and PD-L2 (24). In this study soluble levels of PD-L1, CTLA-4 and TIM-3 increased in the immediate post-operative setting returning to baseline by week 6. Studies have implicated immunosuppressive roles for these three soluble immune checkpoints sPD-L1, sCTLA-4 and sTIM-3, suggesting that in this context their increase in the immediate post-operative setting may be a reflection of the pleiotropic immunosuppressive effects of surgery.

Soluble PD-L1 decreases IFN-γ secretion by T cells and induces T cell apoptosis (25). Regulatory T cells are a prominent source of soluble CTLA-4, which has potent inhibitory properties suppressing IFN-γ-mediated Th1 immune responses (26). The biological effects of sTIM-3 are unknown however, a study by Ge et al., demonstrated that osteosarcoma patients with higher levels of circulating soluble Tim-3 had relatively lower survival suggesting that the surgery-induced decrease in circulating soluble TIM-3 in this study may be a beneficial effect for patients and highlights the immunostimulatory effects of surgical removal of the tumour (27).

Soluble PD-1 was identified as a good prognostic indicator in EGFR-mutated non-small cell lung cancer patients which may suggest that the surgery-induced decrease in circulating levels of PD-1 in post-operative patients in this study may be detrimental to anti-tumour immunity highlighting the immunosuppressive nature of surgery in the immediate post-operative period (28). Circulating soluble LAG-3 was a good prognostic marker in gastric cancer and positively correlated with CD8^+^ T cell frequency and secretion of IL-12 and IFN-γ in peripheral blood (29). Moreover, a study by Fougeray et al., identified soluble LAG-3 protein as an immunopotentiator for therapeutic vaccines (30). Soluble LAG-3 binds to MHC class II inducing maturation of monocyte-derived dendritic cells *in vitro* and is used as a vaccine adjuvant to induce CD4 Th1 and CD8 T cell responses *in vivo* (30). Collectively this suggests that the surgery-induced downregulation of circulating levels of soluble LAG-3 may be a reflection of surgery-induced immunosuppression. Interestingly, in this study patients with higher levels of circulating soluble LAG-3 in the post-operative setting (POD 3) had a better response to neoadjuvant treatment determined by the Mandard pathological scoring system (TRG) (**Supplementary Figure 1**).

Chemotherapy and radiation therapy (RT) are standard therapeutic modalities for patients with esophageal cancers and can induce forms of immunogenic cell death (31). In the current study, intriguingly there is an increase in both DAMPS, calreticulin and HMGB1 in all three compartments in the immediate post-operative period, which would suggest an anti-tumour response however, paradoxically in a study on patients undergoing cytoreductive surgery, increased plasma levels of DAMPs were associated with immune suppression and postoperative infections in patients undergoing cytoreductive surgery (32, 33). Therefore, the concomitant increase in soluble checkpoints and DAMPS may represent the perfect timing for administration of immune checkpoint blockade to offset the prevailing immunosuppressive milieu.

This current study suggests an immunosuppressive milieu perioperatively with reduced Th1 cytokines in the immediate post-operative setting compared to pre-operative levels, upsetting the equilibrium through surgical stress and excision of the tumour burden with a reduction in IFN-γ, IL-12p40, CD28, CD40L and TNF-α. In addition, IP-10 (CXCL-10) which is an important chemokine ligand in recruiting anti-tumor Th1 cells and polarising the immune response to a Th1 phenotype, is significantly reduced perioperatively. There is a simultaneous increase in Th2 pro-tumor cytokines in the immediate post-operative setting, with a significant increase in IL-4, IL-10, IL-16, IL-1RA and MCP-1 before returning to preoperative levels at week 6. Immunosuppressive IL-10 inhibits the differentiation and activation of DCs, which are key activators of anti-tumor effector cells of the adaptive immune system, including cytotoxic CD8^+^ T cells (34). Notably, this switch from a Th1 tumor-suppressive phenotype, which aids cytotoxic CD8^+^ T cells in tumor rejection, to a Th2 tumor-promoting “regulatory” phenotype, which blocks CD8^+^ T-cell activity, is a characteristic outcome in the inflammatory, immune-suppressive tumor microenvironment (35). Myeloid-derived suppressor cells and macrophages recruited to the tumor microenvironment from the bone marrow by tumor cells and Tregs are also potent suppressors of anti-tumor immunity, when they are converted to an immunosuppressive phenotype by cytokines such as IL-10, which was found to increase perioperatively in our study and TGF-β (36) which are secreted by tumor tissue as well as other immune and stromal cells to promote recruitment and suppression of many immune cell types (37).

Interestingly, in parallel to the switch in Th1 to Th2 immunity observed in systemic circulation we also observed a concomitant increase in the levels of circulating soluble PD-L1 and significant decrease in circulating levels of PD-L2. PD-L1 plays an important role in dampening Th1 immune responses whereas PD-L2 is has been shown to play a specific role in dampening Th2 responses (38). Collectively, these findings suggest that soluble PD-L1 and PD-L2 might play an important role in regulating the wound healing responses triggered by surgical excision, which is often characterised by a switch from pro-inflammatory immune responses to an anti-inflammatory immune response (14).

Specific chemokine ligands MIP-1α and MIP-1β, which recruit pro-inflammatory tumour-promoting myeloid immune cells (39), increased in the immediate post-op setting returning to baseline by week 6 and may represent a systemic pro-tumorigenic niche in the immediate post-operative period. In addition to this, high levels of MIP1-α on day 3 correlated with lymphatic invasion (**Supplementary Figure 1**). MIP1-α has been implicated in promoting tumour metastasis to lymph nodes in oral squamous cell carcinoma (40). In addition, MIP1-α induced migration of MCF-7 breast cancer cells *in vitro* (41). Considering the findings of this study in the context of the existing literature MIP1-α may play an important role in promoting metastasis to lymph nodes in the immediate post-operative setting in OAC patients.

Metastatic disease is the leading cause of death among cancer patients and involves a complex and inefficient process, with a high rate of recurrence in oesophageal cancer, especially in the first 18 months (7, 42). Every step of the metastatic process can be rate limiting and is influenced by non-malignant host cells interacting with the tumor cell. Over a century ago, experiments first indicated a link between the immune system and metastasis (43). There is a significant increase in CD276, an immune checkpoint molecule post operatively with its peak at week six. CD276 is aberrantly overexpressed in many types of cancer, and such upregulation is generally associated with a poor clinical prognosis. There is also evidence to indicate an intricate role for CD276 (B7H3) in promoting carcinogenesis and metastasis (44, 45). VEGF-A and PIGF, which are major pro-angiogenic factors associated with cancer angiogenesis and are pro-tumorigenic and Flt-1, also known as vascular endothelial growth factor receptor 1 (VEGFR-1), is a high-affinity tyrosine kinase receptor for VEGF involved in tumor growth and metastasis (46). PIGF/Flt-1 signalling is integral in colorectal cancer progression through increasing the phosphorylation of p38 MAPK, thereby upregulating MMP9 expression; resulting in increasing cellular migration/invasion (47). There is an initial increase in these markers in the immediate post-operative setting which may represent a systemic pro-metastatic niche, which might be priming distal organs for metastatic dissemination and/or promoting the growth of micrometastatic deposits into overt secondary tumors. This is particularly telling in the fact that four of the cohort have already developed metastatic disease. VEGF-A circulating levels positively correlated with lymphatic invasion on day 3, with levels of circulating FLT-1 on day 1 positively correlating with a poor TRG. Importantly, three of those patients who developed metastatic disease had the highest expression of soluble PD-1, PD-L1 and TIM-3 perioperatively with a peak on day 7 and 2 of this cohort had significant increases in angiogenic markers at day 3 and day 7 post operatively.

Our study highlights the prevailing immunophenotype and responses to surgery with a switch in balance towards a Th2 phenotype and consequently, an immunosuppressive milieu. Therefore, shifting the balance in favour of a Th1 phenotype would offer a potent therapeutic approach for reducing tumorigenesis and promoting cancer regression, including potentially radiotherapy and anti-PD-L1/PD-1 strategies. These immunologic consequences of radiotherapy and therapeutic reprogramming of immune responses in tumors, along with how it regulates efficacy and durability to radiotherapy must be explored (48). Consequently, this body of work paves the way for further studies and appropriate trial design are needed to interrogate the use of ICB as a trimodal approach with chemoradiotherapy and chemotherapy alone for locally advanced disease in the neoadjuvant and adjuvant setting to determine the optimal timing and subset of patients for their use in the era of precision targeted therapies.

## Data Availability Statement

No clinical data pertaining to patients will be made available. Requests to access the datasets should be directed to Noel Donlon donlonn@tcd.ie.

## Ethics Statement

The studies involving human participants were reviewed and approved by Tallaght University Hospital/St James’s Hospital Ethics committee. The patients/participants provided their written informed consent to participate in this study.

## Author Contributions

ND, MDa, and AS conducted experiments and contributed to writing the manuscript and were equal contributors. FO’C and MDu contributed to experiments. CH, EM, SR, HT, MM, GC, AB, and CB contributed to sample collection. MC, JO’S, and NR contributed to study design. CD, JR, and JL supervised the study and were responsible for revision of paper. All authors contributed to the article and approved the submitted version.

## Funding

This work was supported by the CROSS Cancer Charity (RCN: 15364).

## Conflict of Interest

The authors declare that the research was conducted in the absence of any commercial or financial relationships that could be construed as a potential conflict of interest.

## Publisher’s Note

All claims expressed in this article are solely those of the authors and do not necessarily represent those of their affiliated organizations, or those of the publisher, the editors and the reviewers. Any product that may be evaluated in this article, or claim that may be made by its manufacturer, is not guaranteed or endorsed by the publisher.

## Supplementary Material

Supplementary Figure 2 Expression of activation markers on CD4 T helper cells **(A)** and CTLs **(B)** in circulation categorised by treatment type. Frequency of CD4 T helper **(C)** and CTL **(D)** differentiation states in circulation categorised by treatment received. Green diamond: no neoadjuvant treatment received (n=1), blue circle: FLOT (n=7), red square: CROSS (n=2) and orange triangle: FOLFOX (n=1).

Supplementary Figure 3 Frequency of circulating cell types categorised by treatment type. **(A)**. Expression of ICs on the surface of circulating CD4 T helper cells **(B)** and CTLs **(C)** as well as expression of DAMPs on CD4 T helper cells **(D)** and CTLs **(E)** categorised by treatment received. Green diamond: no neoadjuvant treatment received (n=1), blue circle: FLOT (n=7), red square: CROSS (n=2) and orange triangle: FOLFOX (n=1).

Supplementary Figure 4 Levels of circulating immunomodulatory and pro-angiogenic mediators categorised by treatment type. Soluble levels of circulating immunostimulatory mediators **(A)** and pro-angiogenic factors **(B)** categorised by treatment received. Green diamond: no neoadjuvant treatment received (n=1), blue circle: FLOT (n=7), red square: CROSS (n=2) and orange triangle: FOLFOX (n=1).
