# Supplementary figures and images for: The Impact of Esophageal Oncological Surgery on Perioperative Immune Function; Implications for Adjuvant Immune Checkpoint Inhibition
Source: Front Immunol. 2022 Jan 27;13:823225. doi: 10.3389/fimmu.2022.823225 (PMC8829578; doi:10.3389/fimmu.2022.823225)

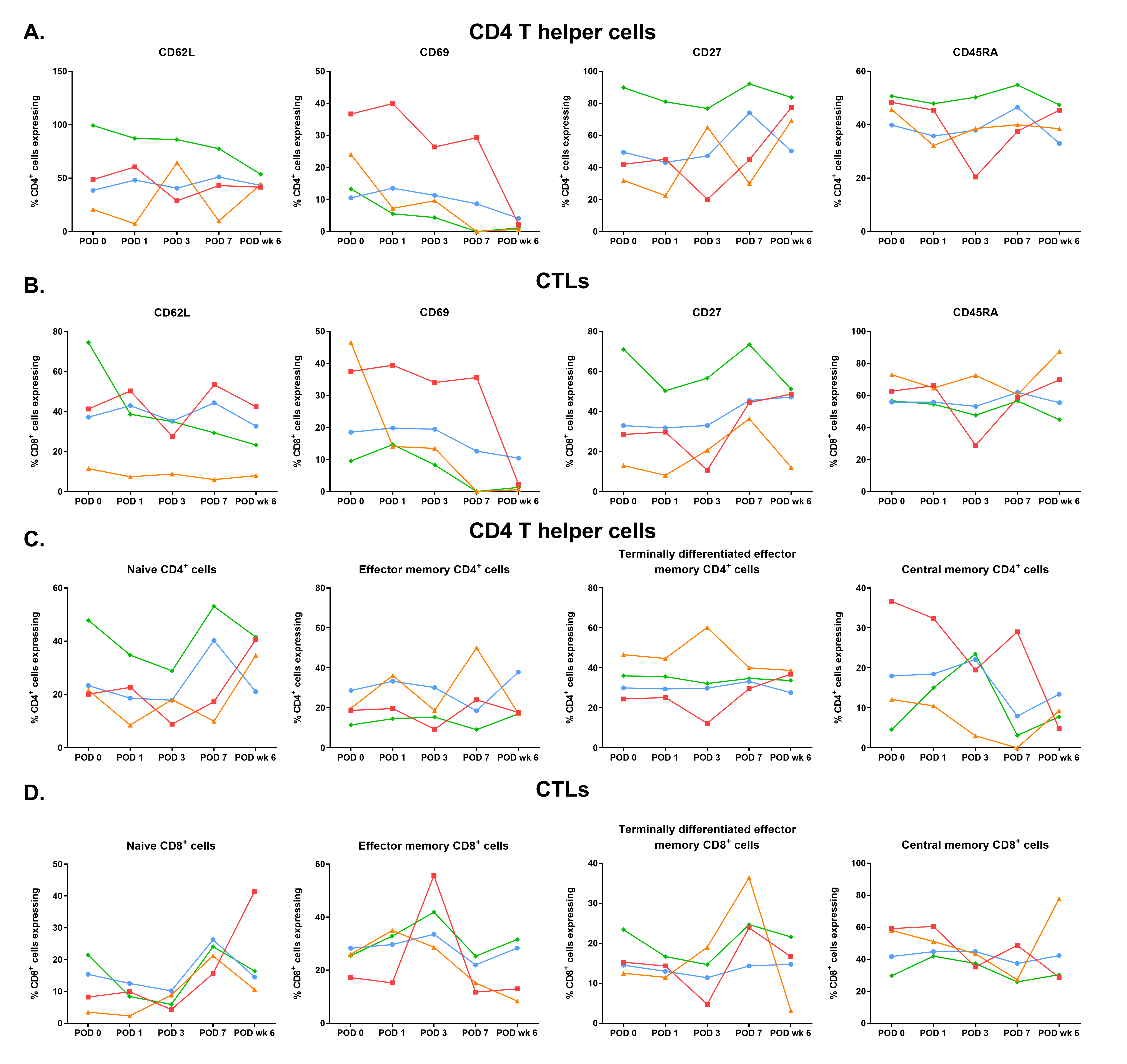

Supplement: Supplementary file 1 [file Image_1.tif]

## Day 0

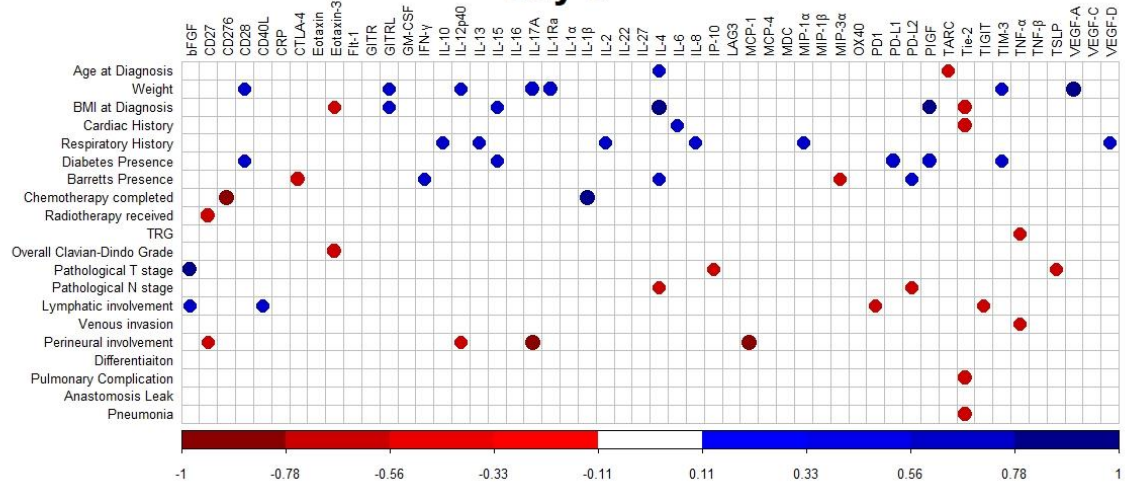

## Day 1

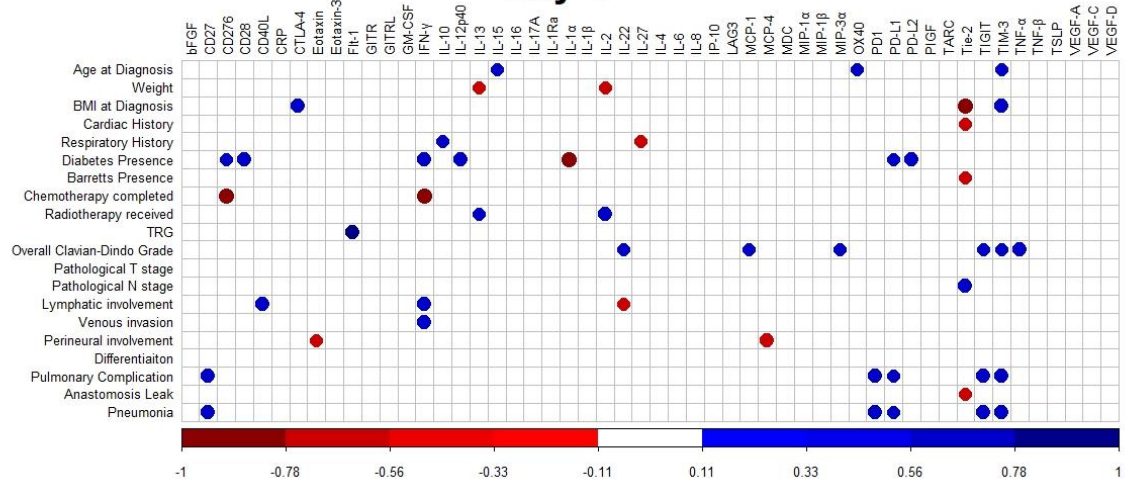

## Day 3

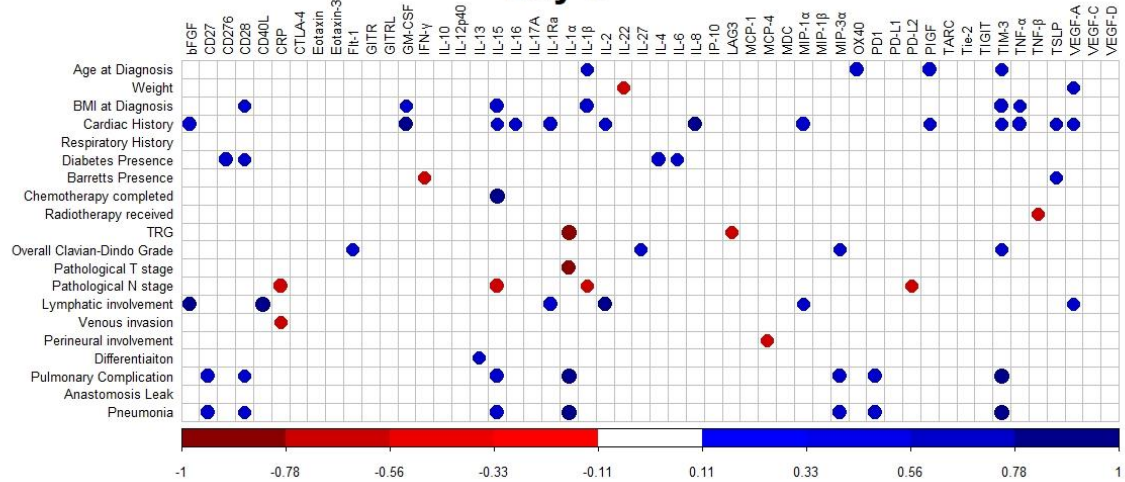

## Day 7

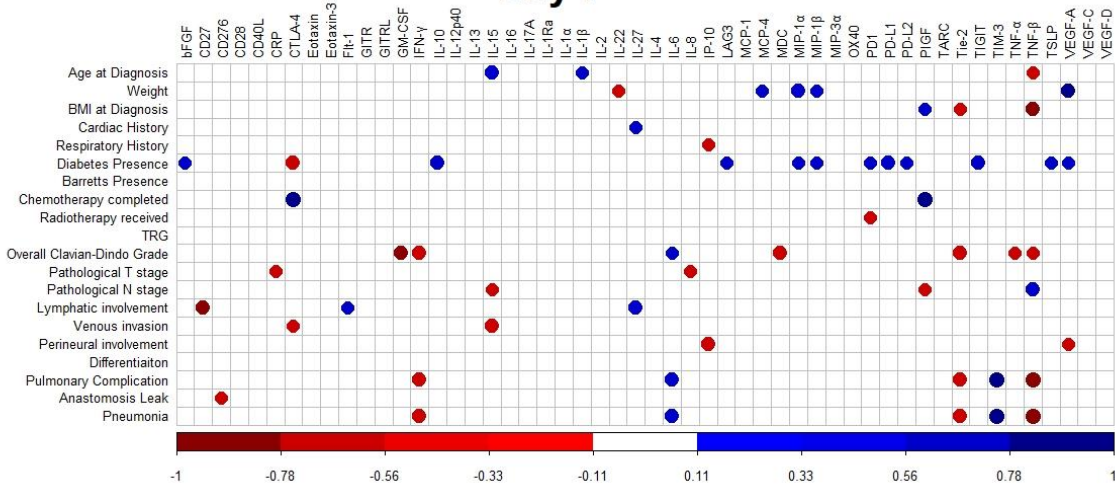

## Day 42

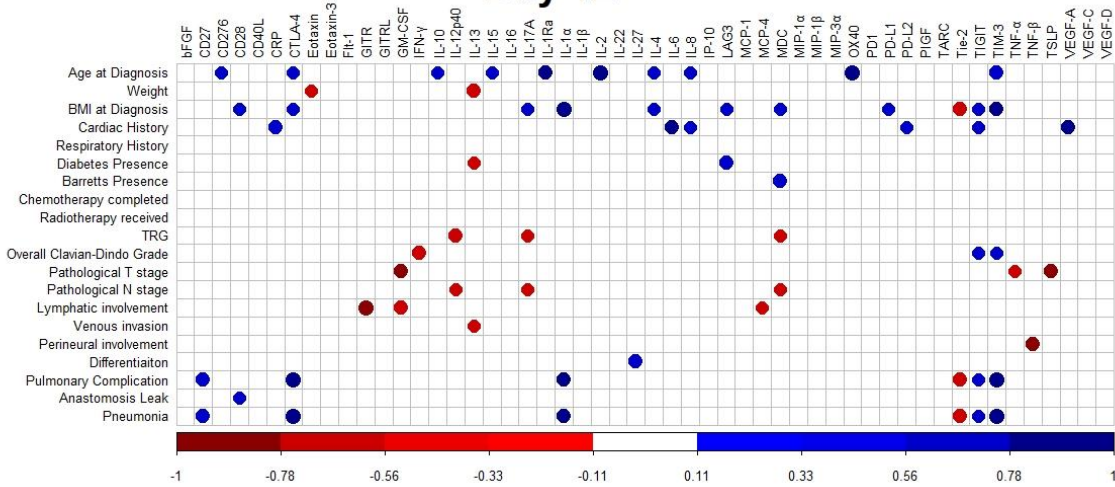

Supplement: Supplementary file 2 [file Table_1.pdf]

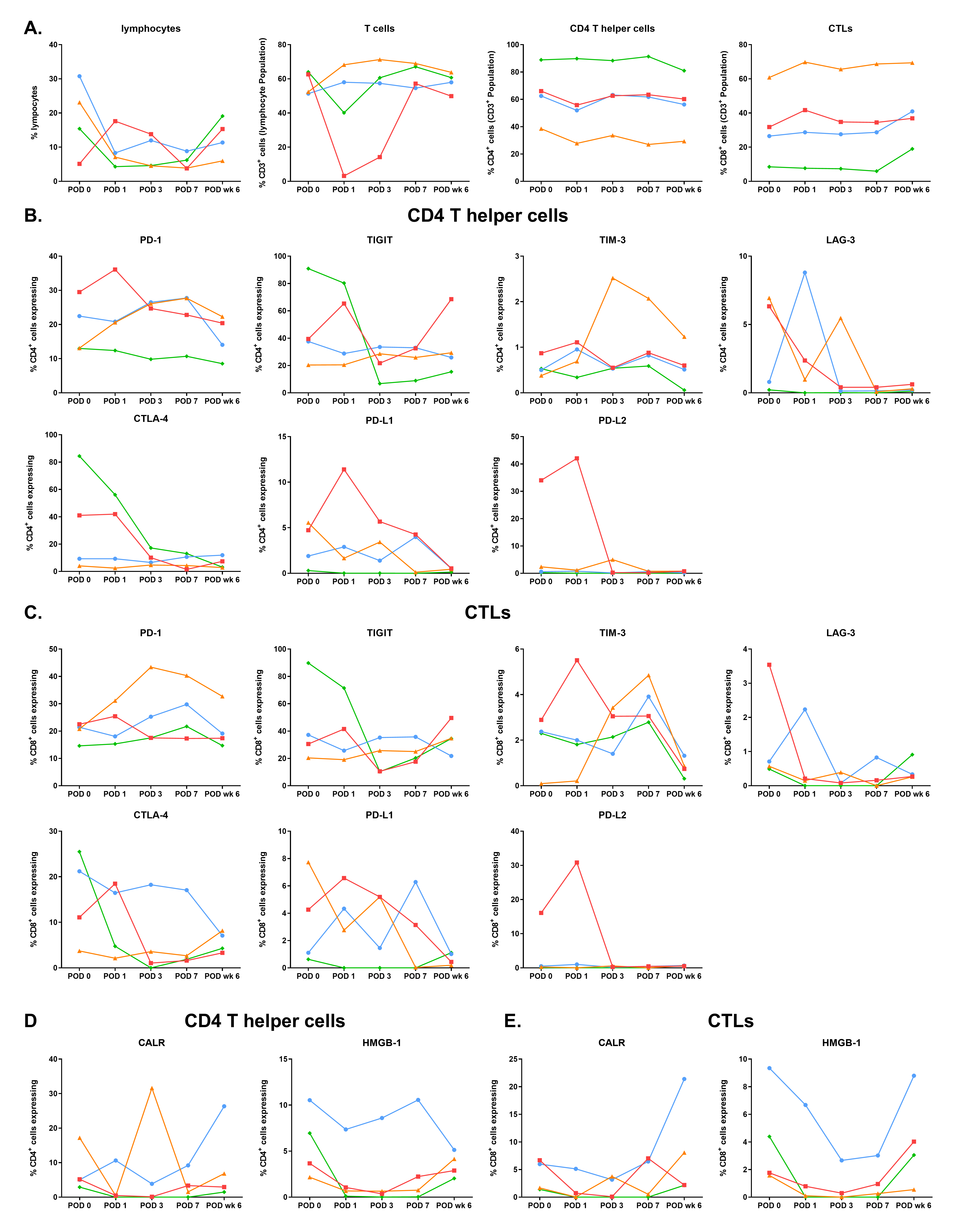

Supplement: Supplementary Figure 3 — Frequency of circulating cell types categorised by treatment type. (A). Expression of ICs on the surface of circulating CD4 T helper cells (B) and CTLs (C) as well as expression of DAMPs on CD4 T helper cells (D) and CTLs (E) categorised by treatment received. Green diamond: no neoadjuvant treatment received (n=1), blue circle: FLOT (n=7), red square: CROSS (n=2) and orange triangle: FOLFOX (n=1). [file Image_3.tif]

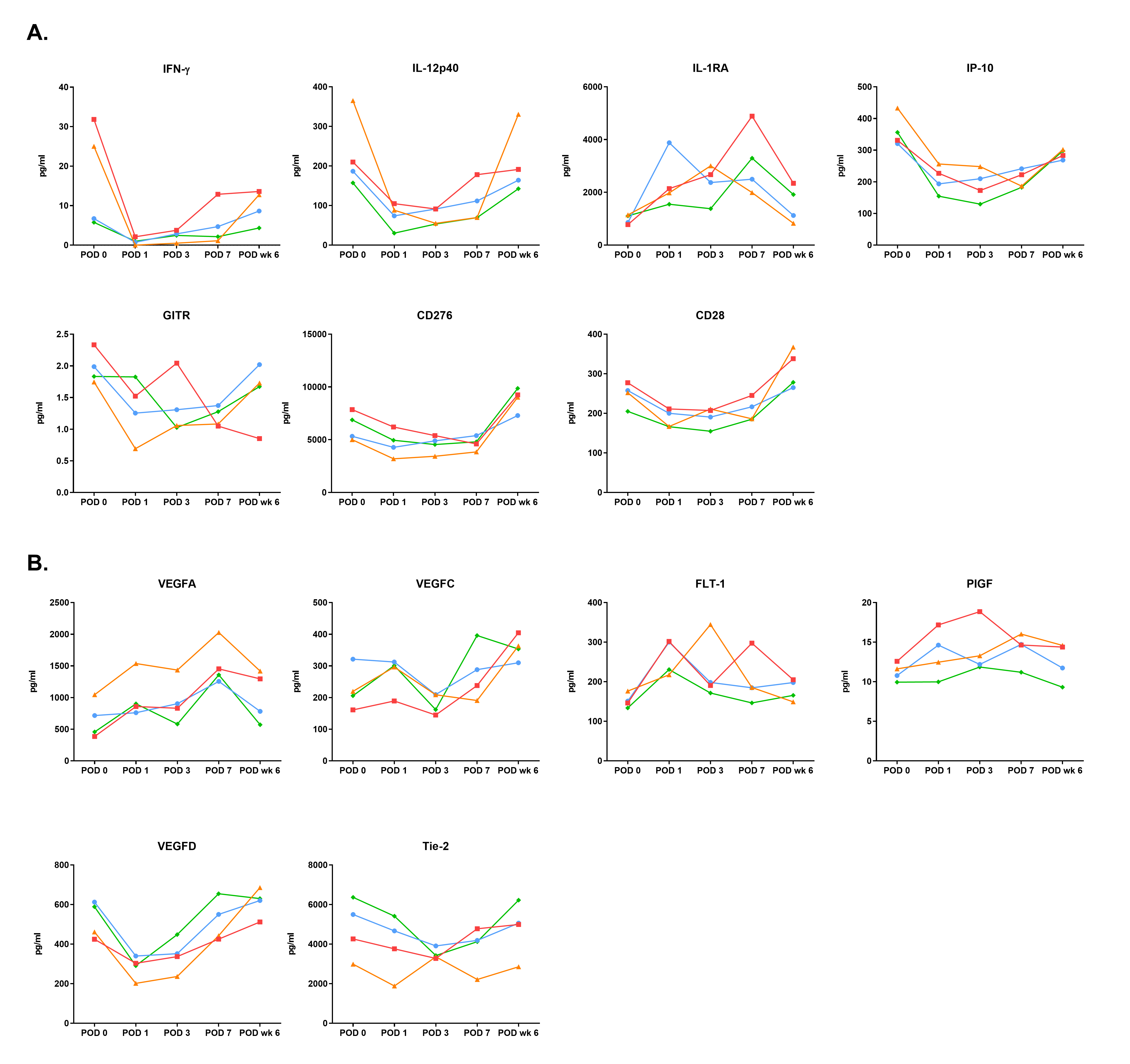

Supplement: Supplementary Figure 4 — Levels of circulating immunomodulatory and pro-angiogenic mediators categorised by treatment type. Soluble levels of circulating immunostimulatory mediators (A) and pro-angiogenic factors (B) categorised by treatment received. Green diamond: no neoadjuvant treatment received (n=1), blue circle: FLOT (n=7), red square: CROSS (n=2) and orange triangle: FOLFOX (n=1). [file Image_4.tif]
